# Structurally Coloured Secondary Particles Composed of Black and White Colloidal Particles

**DOI:** 10.1038/srep02371

**Published:** 2013-08-06

**Authors:** Yukikazu Takeoka, Shinya Yoshioka, Midori Teshima, Atsushi Takano, Mohammad Harun-Ur-Rashid, Takahiro Seki

**Affiliations:** 1 Graduate School of Engineering, Nagoya University, Furo-cho, Chikusa-ku, Nagoya, 464–8603, Japan; 2 Graduate School of Frontier Biosciences, Osaka University, 1–3 Yamadaoka, Suita, Osaka 565–0871, Japan

## Abstract

This study investigated the colourful secondary particles formed by controlling the aggregation states of colloidal silica particles and the enhancement of the structural colouration of the secondary particles caused by adding black particles. We obtained glossy, partially structurally coloured secondary particles in the absence of NaCl, but matte, whitish secondary particles were obtained in the presence of NaCl. When a small amount of carbon black was incorporated into both types of secondary particles, the incoherent multiple scattering of light from the amorphous region was considerably reduced. However, the peak intensities in the reflection spectra, caused by Bragg reflection and by coherent single wavelength scattering, were only slightly decreased. Consequently, a brighter structural colour of these secondary particles was observed with the naked eye. Furthermore, when magnetite was added as a black particle, the coloured secondary particles could be moved and collected by applying an external magnetic field.

Aggregations composed of fine submicron spherical colloidal particles (hereafter referred to as “colloidal particles”) can exhibit a decrease in the density of optical states (DOS) in the visible light range, depending on the states of aggregation and the contrast of the refractive index of the photonic structure^1^,^2^,^3^,^4^,^5^. In many previous reports, the aggregations of colloidal particles were required to possess long-range order and periodicity, *i.e.*, a colloidal crystal structure, to completely suppress the DOS or to exhibit a photonic band gap (PBG). Such an aggregation can exhibit structural colour as a result of the reflection of specific wavelengths of visible light by the PBG. However, it has been recently recognised that colloidal amorphous arrays also show structural colours despite their lack of long-range order. Coloration mechanisms of amorphous systems have been interpreted as the single (and partially double) scattering process of light with the short-range order^6^,^7^,^8^,^9^, while several studies have suggested that there is a significant suppression in the DOS that results in the coloration^10^,^11^,^12^. The coloration characteristics of these aggregations are expected to differ depending on the type of aggregation of the colloidal particles: a colloidal crystal exhibits a glossy and angle-dependent structural colour depending on the viewing and illumination angles, whereas a colloidal amorphous array exhibits a matte and angle-independent structural colour^8^,^13^,^14^,^15^.

In the past two decades, membrane assemblies of colloidal particles have been the subject of extensive studies^16^,^17^,^18^,^19^,^20^,^21^ because of their potential application in energy-saving displays^22^ and sensors^23^, which utilise the optical characteristics of these assemblies. Recently, because of the advancement in technologies for preparing colloidal assemblies, researchers have been able to prepare not only membrane assemblies but also assemblies with various shapes and sizes^24^,^25^. One of the most extensively studied assemblies is the secondary particle formed from colloidal particles, which can be useful for the development of coloured materials used in displays and for biological and chemical screenings^26^,^27^,^28^,^29^,^30^,^31^. Because structurally coloured secondary particles do not contain conventional dyes, they can exhibit a colour that does not fade. Additionally, because the chemical species currently available for products are limited and pose a significant environmental risk, structurally coloured assemblies composed of safer chemicals can be used as environmentally friendly pigments^13^. For example, because the dyes and pigments available for cosmetic products are strictly controlled, structurally coloured materials prepared from safer colloidal particles are expected to be useful as new, colourful pigments.

In this study, we prepared colourful secondary particles by controlling the aggregation states of the colloidal particles and by adding black particles to the aggregations. To create the secondary particles, we used environmentally friendly materials that enabled them to be used as green pigments. Fine colloidal silica particles (hereafter referred to as “silica particles”) were used as colloidal particles, which appear white to the naked eye when the colloidal particles are in random states. The toxicity of silica particles greater than 300 nm in diameter has not been detected *in vivo*^32^. We used carbon black (CB) or magnetite as black particles, both of which are known to be environmentally friendly and biologically harmless pigments.

## Results

**Figure 1:**
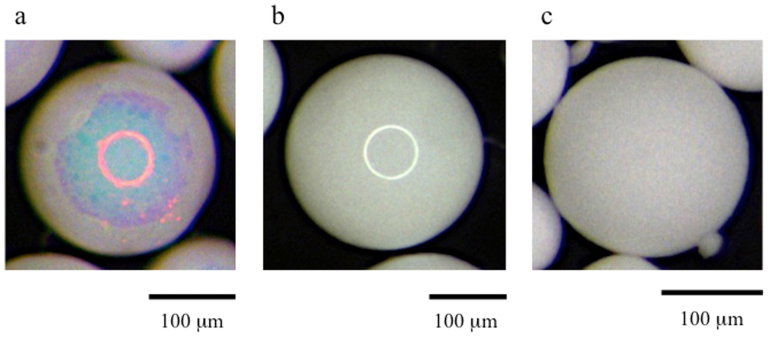
Photographs of the secondary particles. (a) A secondary particle composed of silica particles 360 nm in diameter viewed under a microscope. (b) A secondary particle composed of silica particles 280 nm in diameter viewed under a microscope. (c) A secondary particle composed of silica particles 360 nm in diameter prepared with the addition of NaCl.

First, we describe the appearance of the secondary particles composed of only silica particles. To prepare the secondary particles in a typical experiment, drops of an aqueous suspension of the silica particles (with diameters of 280 or 360 nm) were added to a stirred oil phase at 60°C in a round-bottom flask using a microsyringe^30^. After the reaction had proceeded for more than 12 hours, we allowed the mixture to reach room temperature before collecting the sediments. The sediments were washed using n-hexane and then dried in an oven. Subsequently, dried and spherical secondary particles, composed of the silica particles with a coefficient of variation of 15%, were obtained. The average size of the secondary particles can be changed from 100 to 500 μm by varying the inner diameter of the needle connected to the microsyringe and the drip rate of the suspension under our experimental conditions. Glossy secondary particles were obtained when pure water was used as a dispersion medium for the suspension (Figs. 1a and 1b). The surface of the glossy secondary particles was sufficiently smooth to permit a ring-shaped light to be reflected through a microscope using white light. The ring-shaped specular colour appeared red when silica particles 360 nm in diameter were used. The glossy secondary particles composed of silica particles 280 nm in diameter also exhibited the same optical property, and the ring-shaped specular colour appeared faint green. However, the secondary particles prepared using an aqueous suspension of silica particles and NaCl exhibited a colour that appeared matte and white, irrespective of the size of the silica particles (Fig. 1c). In pure water, the silica particles likely form a non-close-packed crystal structure due to the repulsive force between the electric double layers of each particle when the aqueous suspension is concentrated in hot oil^33^. Consequently, a close-packed crystal structure similar to the non-close-packed crystal structure is formed after drying. The structural colour must result from the occurrence of the p-PBG due to the formation of the crystal structure composed of the silica particles^34^. In contrast, the thickness of the electric double layer of the silica particles decreases when NaCl, which acts as an electrolyte in water, is added to the aqueous suspension; consequently, the repulsive force between the silica particles diminishes. Therefore, the silica particles are flocculated by the evaporation of water. Thus, it appears that the silica particles form an amorphous array in the dried state of the secondary particles. Although the amorphous array of silica particles can reveal wavelength-specific constructive reflection, observing the structural colour from the relatively thick amorphous array is difficult because of the large contribution of incoherent multiple scattering^13^,^14^,^35^.

**Figure 2:**
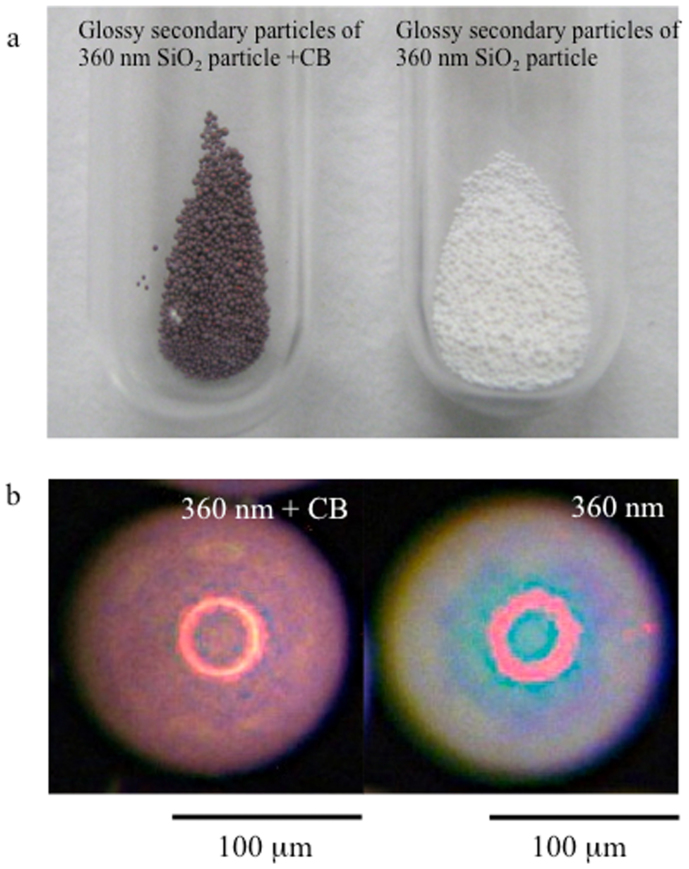
Photographs of the glossy secondary particles with and without CB. (a) Secondary particles composed of silica particles 360 nm in diameter and CB (left). Secondary particles composed of silica particles 360 nm in diameter (right). (b) A secondary particle composed of silica particles 360 nm in diameter and CB (left). A secondary particle composed of silica particles 360 nm in diameter (right).

**Figure 3:**
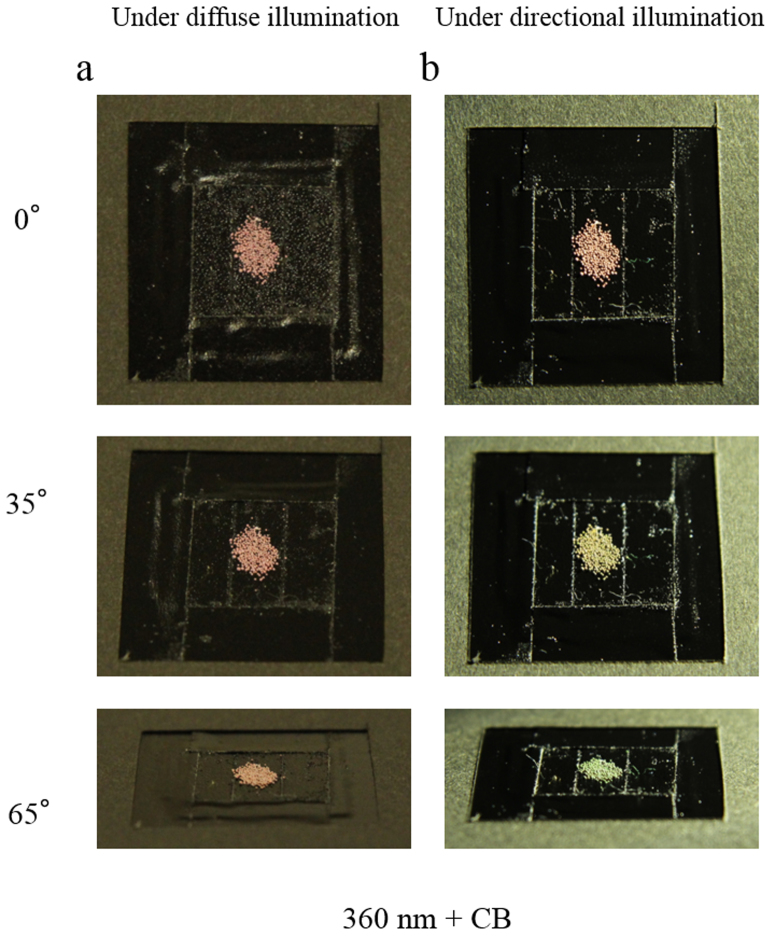
Photographs of the glossy secondary particles with CB under different light conditions. (a) Secondary particles composed of silica particles 360 nm in diameter and CB viewed under diffused light on an 18 mm × 18 mm cover glass. (b) The same sample with (a) viewed under directional light.

**Figure 4:**
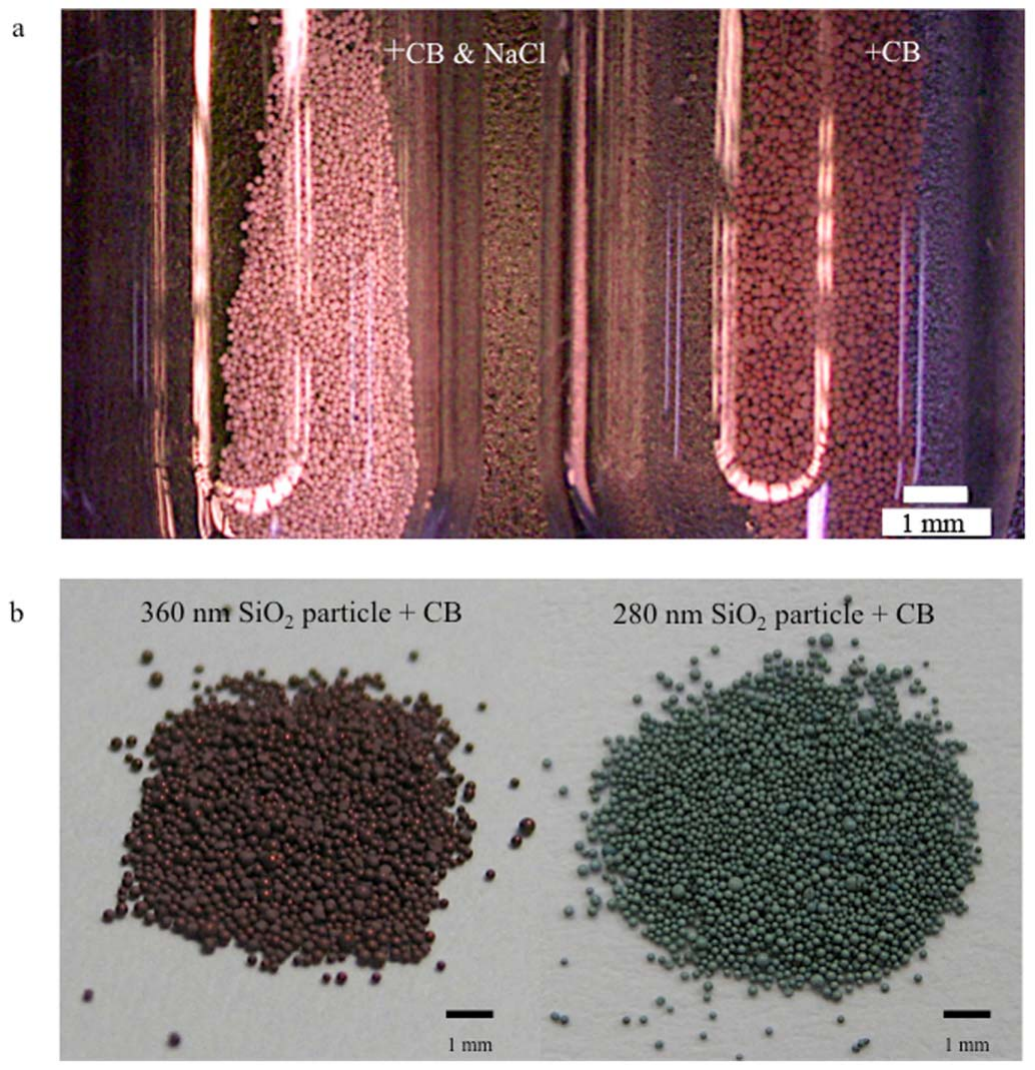
Photographs of the secondary particles with CB prepared with and without NaCl. (a) Secondary particles composed of silica particles 360 nm in diameter and CB, which were prepared with and without NaCl. b) Secondary particles composed of silica particles 360 nm in diameter and CB (left). Secondary particles composed of silica particles 280 nm in diameter and CB (right).

Colloidal crystals and colloidal amorphous arrays composed of colloidal particles containing a small number of black particles, such as CB, are known to exhibit bright structural colours due to the reduction in the incoherent scattering, which is due to the addition of the black particles^13^,^14^. In this study, a small amount of CB was first introduced into the secondary particles. Fig. 2a presents an optical image of the glossy secondary particles primarily composed of 360-nm-diameter silica particles, both with and without the addition of CB. The glossy secondary particles with the CB appear red in colour, whereas the glossy secondary particles without the CB appear white to the naked eye. When observed with a microscope, the glossy secondary particles with the CB exhibit a completely red colour; additionally, a specularly reflected, ring-shaped light is clearly observed that appears red in colour (Fig. 2b). These observations indicate that there are two reflection mechanisms causing the red colour of the secondary particles: a diffuse reflection and a specular one. Interestingly, we have found that the lighting method has a large effect on the angular dependence. Figs. 3a and 3b show the colour behaviours of the glossy secondary particles with the CB on a flat, black board depending on the lighting and the viewing angle. The samples were observed at various angles relative to the normal position of the planar surface of the black board. Under diffuse lighting, the colour from the glossy secondary particles with the CB only slightly changes with the viewing angle (Fig. 3a), whereas under directional lighting, the colour from the glossy secondary particles with the CB drastically changes with the viewing angle (Fig. 3b). Fig. 4a presents an optical image of the secondary particles prepared from the aqueous suspensions of the silica particles 360 nm in diameter with both CB and NaCl (left) compared with an image of the secondary particles prepared with only CB (right). The secondary particles obtained using both CB and NaCl also exhibited a bright red colour, although the red colour was somewhat pale and matte. Additionally, because the position of the peak in the reflection spectra from the secondary particles can vary depending on the size of the silica particles, we obtained differently coloured secondary particles using silica particles of different sizes (Fig. 4b). We know that both the single coherent scattering, which is wavelength-selective, and the multiple scattering of light throughout the entire visible region contribute to the optical properties of amorphous arrays. When CB was incorporated into the secondary particles, the multiple scattering of light was considerably reduced, whereas the reflectance peak intensity caused by the single scattering was only slightly decreased. Consequently, the saturation of the structural colour of the secondary particles is sufficiently enhanced that it can be observed with the naked eye.

**Figure 5:**
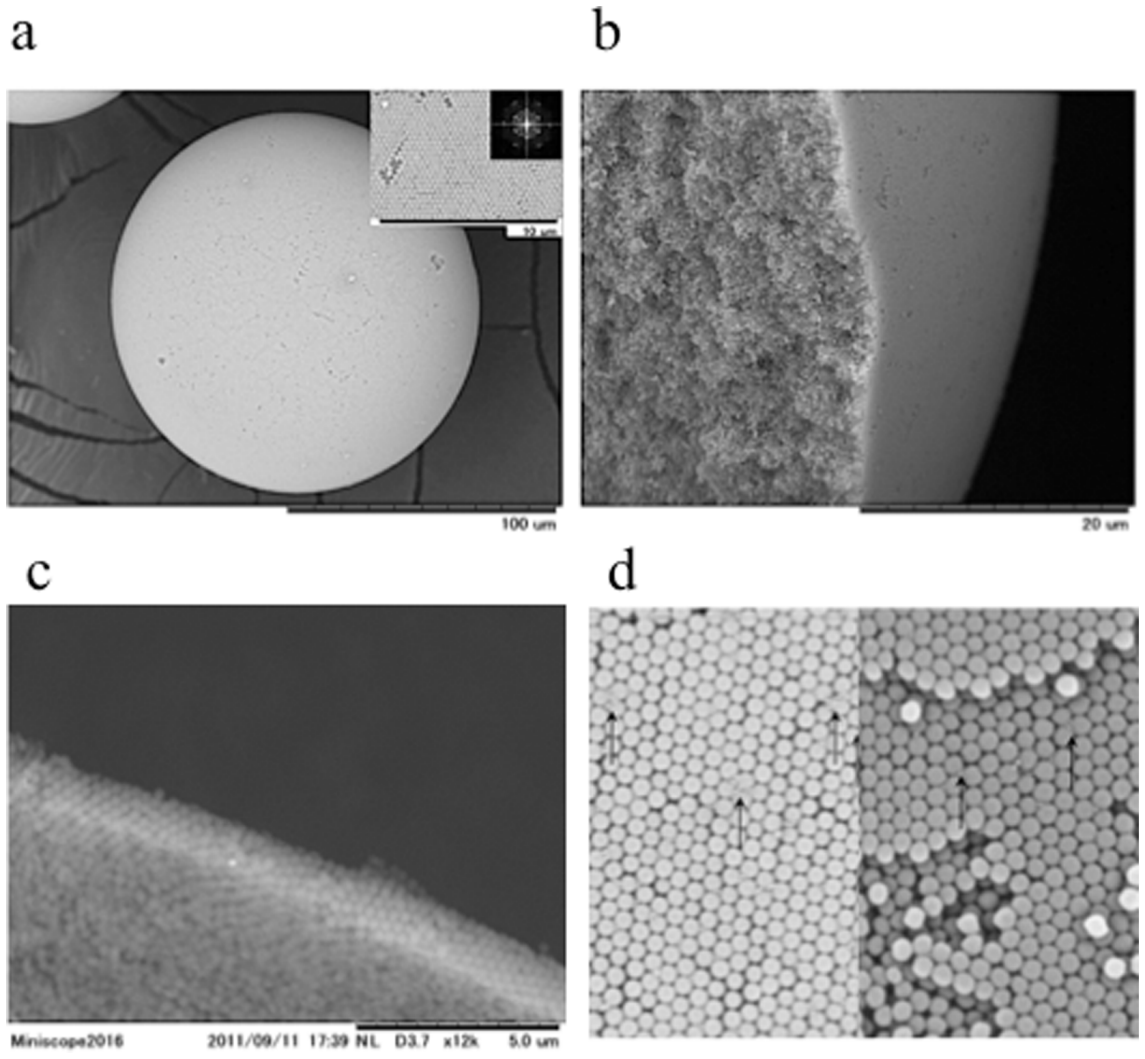
Microstructures of the glossy secondary particles. (a) An SEM image of a glossy secondary particle composed of 360-nm silica particles. The inset presents a magnification of the SEM image and the FT image. (b) The cross-sectional SEM image of the glossy secondary particle prepared by grinding the glossy secondary particle in liquid nitrogen. (c) A cross-sectional SEM image of a glossy secondary particle prepared by microtoming the embedded sample. (d) SEM image of the surface of a glossy secondary particle. The CB is indicated by arrows.

To investigate the microstructures, we first observed the aggregation states of the silica particles in the secondary particles using a scanning electron microscope (SEM). Fig. 5 presents SEM images of the glossy secondary particles. The ordered colloidal crystal structure of the silica particles can be observed on the surface of a glossy secondary particle (Fig. 5a). This secondary particle, which has an ordered crystal structure, has been studied by many groups and is known as the “Photonic Ball”^30^. The inset in Fig. 5a presents a Fourier transform (FT) image that has six-fold symmetry; the aggregation of the silica particles results in a colloidal crystal with long-range order and periodicity. There are grain boundaries with sizes ranging from a few micrometres to a few dozen micrometres in the colloidal crystal region, which are similar to those found in membrane colloidal crystals. However, based on the cross-sectional images presented in Figs. 5b and 5c, the silica particles form a random arrangement inside the secondary particles while forming a crystalline structure on the surface. Although the thickness of the crystalline structure varies in different regions, the silica particles form crystalline structures that range in thickness from six to ten layers. Based on this structural information, we conclude that there are two mechanisms associated with the coloration of the glossy secondary particles, as indicated in the previous paragraph. The first mechanism is the development of colour by Bragg reflection caused by the crystal arrangement on the surface, and the second is the development of colour by the coherent single scattering from the arrangement of the amorphous array. Fig. 5d presents an SEM image that reveals the surface and interior of a glossy secondary particle that includes a small amount of CB. We observe that the CB is dispersed throughout the secondary particle. Therefore, we confirmed that CB can be uniformly dispersed in the secondary particle using our preparation procedure.

**Figure 6:**
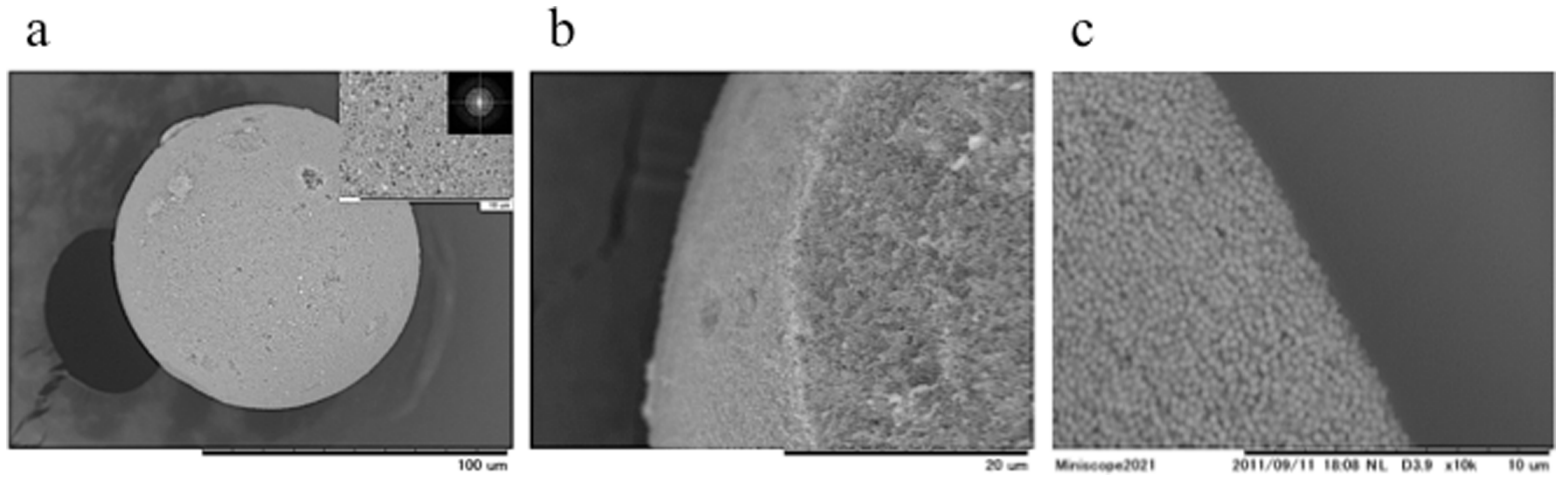
Microstructures of the matte secondary particles. (a) An SEM image of a matte secondary particle composed of 360-nm silica particles. The inset presents a magnification of the SEM image and the FT image. (b) A cross-sectional SEM image of a matte secondary particle prepared by grinding the matte secondary particle in liquid nitrogen. (c) A cross-sectional SEM image of the matte secondary particle prepared by microtoming the embedded sample.

In contrast, we observed that the silica particles form a uniform and random arrangement in the matte secondary particles, irrespective of the surface and interior structures (Fig. 6). The circular ring pattern in the FT image, shown in the inset of Fig. 6a, indicates that the microstructure of the aggregations composed of silica particles is isotropic and has short-range order. We were unable to observe any differences in the arrangement of the silica particles between the surface area and interior of the secondary particles in the cross-sectional images (Figs. 6b and 6c). As demonstrated above, we confirmed that the silica particles form an amorphous array in the matte secondary particles obtained by adding NaCl to the aqueous suspension.

**Figure 7:**
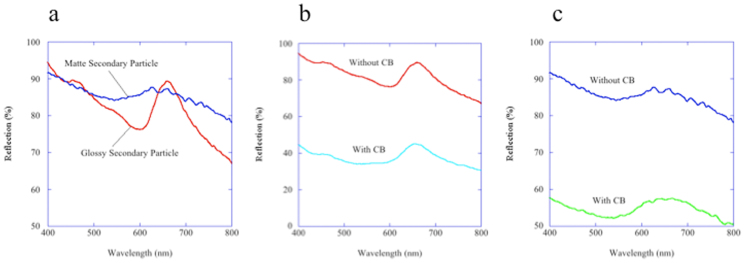
Reflection spectra of the secondary particles. (a) Reflection spectra of the glossy and matte secondary particles composed of 360-nm silica particles. (b) Reflection spectra of the glossy secondary particles composed of 360-nm silica particles with and without CB. (c) Reflection spectra of the matte secondary particles composed of 360-nm silica particles with and without CB.

The reflection spectra of the secondary particles were measured and provide information about the internal microstructure of the secondary particles. Fig. 7a presents the reflection spectra of the glossy and matte secondary particles composed of silica particles 360 nm in diameter. For the glossy secondary particles, we observed a peak wavelength, *λ*_max_, of 660 nm and a half bandwidth, Δ*λ*, of approximately 80 nm. The value of Δ*λ*/ *λ*_max_ is 0.121, which is larger than the value of 0.049 that was theoretically obtained for the (111) plane of a face-centred cubic opal crystal, the most energetically stable structure^36^. This difference may be due to the limited number of crystalline layers or to the imperfect arrangement of the primary particles. In contrast, a *λ*_max_ of 645 nm was observed in the reflection spectrum of the matte secondary particle. The half bandwidth, Δ*λ*, of the peak of the matte secondary particle was considerably broader than that observed for the peak of the glossy particle, which is a characteristic optical property for a colloidal amorphous array^35^,^37^. Although wavelength-specific constructive reflection was observed from both types of secondary particles in the reflection spectra, strong light scattering in the entire visible region was also observed in both spectra. This strong light scattering is incoherent multiple scattering by individual particles inside the secondary particles. Because the incoherent light scattering from these secondary particles is significant across the entire visible region, both types of secondary particles appeared white to the naked eye. However, the contribution of the coherent single scattering became more prominent when the incoherent multiple scattering was reduced due to the addition of the CB, as shown in Figs. 7b and 7c^13^,^14^,^38^,^39^. Consequently, the structural colours due to the pronounced wavelength-specific constructive reflection of these secondary particles became more saturated.

**Figure 8:**
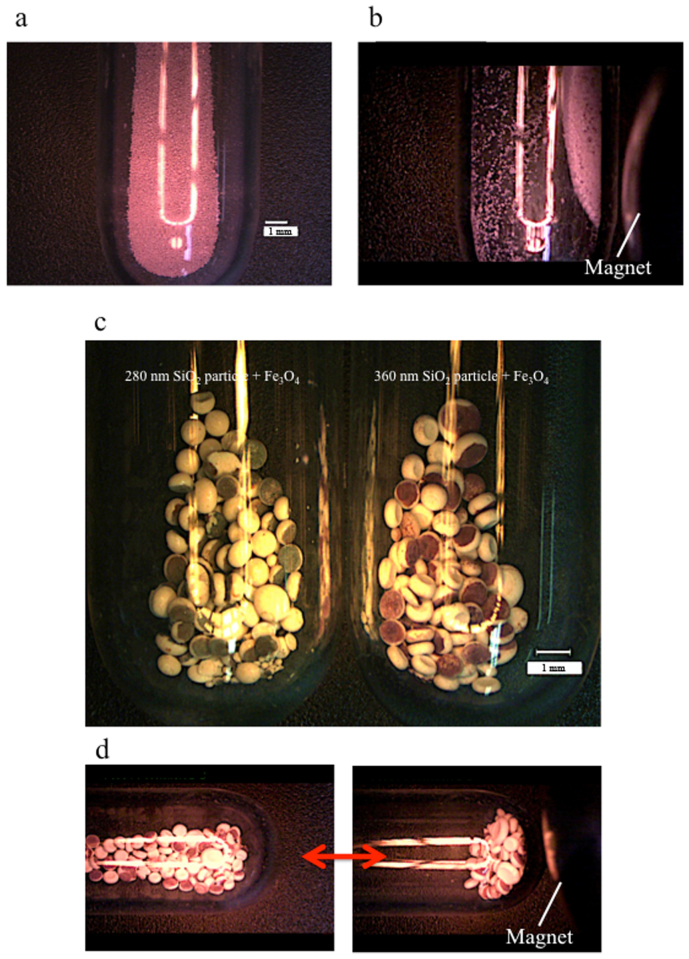
Photographs of the secondary particles with magnetite. (a) Secondary particles composed of 360-nm silica particles with the addition of magnetite. (b) The secondary particles, composed of 360-nm silica particles with the addition of magnetite, were collected by applying an external magnetic field. (c) Flattened Janus secondary particles, characterised by one white side and one red side, composed of 280-nm silica particles (left) and 360-nm silica particles (right). (d) Flattened Janus secondary particles composed of 360-nm silica particles face the same direction in the presence of an external magnetic field.

In addition, we prepared secondary particles from the silica particles containing magnetite as a black particle to obtain colour pigments with variable performances. Magnetite is a commonly used, non-toxic and environmentally friendly black particle. Fig. 8a shows a secondary particle prepared using a suspension of silica particles 360 nm in diameter and a small amount of magnetite. This secondary particle also exhibits a bright red colour. Because magnetite is magnetic, we can move and collect the coloured secondary particles using an external magnetic field (Fig. 8b and Movie 1). If we place the larger droplets of the aqueous solution, which are approximately a few millimetres in diameter and include the silica particles and a small amount of magnetite, into oil at 60°C during the preparation of the secondary particles, the heaviest magnetite (5.2 g/cm^3^) accumulates on the bottom of the droplet before drying. Consequently, we obtained flattened Janus secondary particles, in which one side is red and the other side is white (Fig. 8c). Using 280-nm-diameter silica particles, the hue of the coloured side of the flattened Janus secondary particles can be changed (Fig. 8c). Because the coloured portion contains magnetite, the Janus secondary particles face the same direction in the presence of an external magnetic field (Fig. 8d and Movie 2). The skin colour of fish can generally change through the active concentration or dispersion of pigment granules in the interior region of the pigment cell. The analogous colour change in artificial materials may be achieved using stimuli-responsive structurally coloured pigments.

## Discussion

We observed two mechanisms that are associated with the wavelength-specific constructive reflection in the secondary particles of this study. The first mechanism is Bragg reflection, which results from the crystalline structure of the silica particles. The second mechanism is coherent single scattering by the amorphous array of the silica particles. Because these wavelength-specific constructive reflections exhibit different angular dependences, these particles appear as different colours under various lighting conditions. For example, using a directional light and changing the viewing angle, iridescence from the glossy secondary particles can be observed because of the coloration mainly caused by the Bragg reflection (Fig. 3b). In contrast, under diffuse lighting, the change in the structural colour of the glossy secondary particles with different viewing angles is very small because the coloration caused by the amorphous structure is more significant under this lighting condition (Fig. 3a). Therefore, in the case of the matte secondary particles, only the coherent single scattering mechanism contributes to the coloration. If we create fine secondary particles with various sizes, the colour appearance may also be changed. As noted above, the coloration of the secondary particles is affected by multiple factors, including the size of the primary particle, the aggregation state, the number of crystalline layers, the method of illumination, the viewing angle, and, most likely, the size of the secondary particle. The key to obtaining colourful pigments with an appropriate colour is the careful control of these factors.

**Figure 9:**
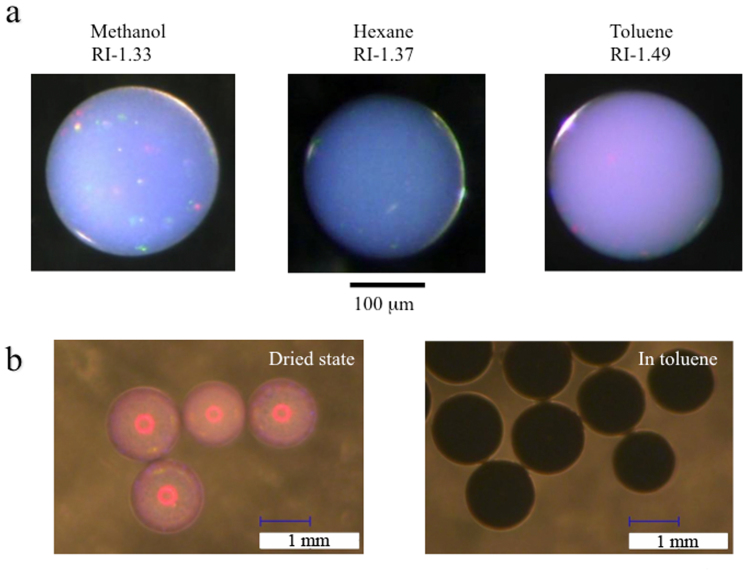
Photographs of the glossy secondary particles under solvents. (a) Secondary particles composed of 360-nm silica particles in different solvents. (b) Secondary particles composed of 360-nm silica particles with CB in the dried state and in toluene.

If the prepared secondary particles are used as pigments in some solvents, the colours can differ from those in air due to the different refractive indices. We know that colloidal amorphous arrays exhibiting good colour saturation as a result of decreasing refractive index contrast can be achieved^31^. For example, our glossy secondary particles displaying a whitish colour in the dried state exhibit good saturation of colour in solvents (Fig. 9a) because of a reduction in the refractive index contrast. Moreover, it is expected that the wavelength becomes longer because the optical length is increased. The magnitude of reflectance will also be decreased due to the smaller refractive index contrast. Therefore, to realise the desired optical properties, it will be necessary to adjust the size of the primary particles, the number of crystalline layers, and the size of the secondary particles. Meanwhile, the glossy secondary particles with black components exhibit black colour in solvents (Fig. 9b), whereas the secondary particles display a vivid colour in the dried state. It may be possible to coat the secondary particle with a protective layer that prevents a solvent from penetrating into the interior of the secondary particle. In this case, similar optical properties to those observed in air are expected. Such an approach is now under investigation.

In conclusion, we observed that we can change the aggregation states of secondary particles composed of silica particles, which were prepared by placing droplets of a suspension containing silica particles into hot oil followed by drying, with or without NaCl. A glossy secondary particle is obtained in the absence of NaCl, whereas a matte secondary particle is obtained in the presence of NaCl. The addition of a black particle, such as CB or magnetite, to the aqueous suspension of the silica particles results in a brighter structural colour of the secondary particles. Because all of the materials used to prepare these structurally coloured secondary particles are non-toxic and environmentally friendly, we expect that these secondary particles can be useful as green pigments. Additionally, with the addition of stimuli-responsive particles, such as magnetite, we can prepare highly functional “colourful balls”.

## Methods

### Materials

We used an aqueous suspension containing 25 wt% of the silica particles. The silica particles used in this study were 280 and 360 nm in diameter. The CB used in this study had an average particle diameter of 100 nm and had carboxyl groups on the surface, enabling it to be dispersed in water. The average diameter of the magnetite was 10 nm, and it was used in conjunction with a dispersing agent. The concentration of NaCl in the suspension was 0.1 mol/L. To obtain the secondary particles composed of the silica particles and the CB, we used silicone oil with a kinetic viscosity of 100 cSt at 25°C. Hexadecane oil was used to prepare the secondary particles that were composed of the silica particles and the magnetite.

### Preparation of Secondary Particles

A typical experimental procedure for preparing the secondary particles is described as follows. Tiny droplets of the aqueous suspension of the silica particles were obtained using a microsyringe and a needle with an inner diameter of 1 mm. These droplets were placed into 800 mL of hot oil at a rate of 1 mL/min at 60°C; the oil was stirred in a 2-L, round-bottomed flask with a stirrer. After stirring for 12 h, the water in the droplets had completely evaporated. After the recovered sedimented product was repeatedly washed with hexane and dried in a thermostat bath, we obtained secondary particles composed of silica particles.

### Characterisation

Photographs evidencing the structural colours of the samples were collected using a digital camera and a digital microscope (KEYENCE VHX-500). Angular dependence was also examined under two types of illumination. In the first type (diffuse illumination), the illuminating light did not come from a specific direction; the sample was illuminated by several ceiling lights and by the secondary scattering from the surrounding walls. In the second type, a fibre optic illuminator (Olympus, LG-PS2) was employed to realise directional illumination. The illuminating light came from a direction tilted by approximately 50 degrees from the normal direction of the sample surface, and the tilting direction was nearly along the upper direction of the photographs in Fig. 3. The arrangement of the silica particles in the secondary particles was investigated using a scanning electron microscope (Hitachi, Miniscope TM3000). To observe the cross section of the secondary particles, we prepared samples using two methods as follows. In the first method, we placed the secondary particles in a pot containing liquid nitrogen and gently ground them. In the second method, the secondary particles were first embedded in a polymer, and an ultramicrotome was then used to obtain ultra-thin sections. These samples were coated with a 10-nm Au-Pd layer, and images were obtained using an SEM operated at 15 kV. The 2D Fourier power spectra of the SEM images were obtained using image analysis software (Image Pro). An Ocean Optics USB2000 fibre optic spectrometer and a UV-Vis spectrometer (Nippon Bunko Company, V-670) with an absolute reflectance measurement unit (ARMN-735) were also used to measure the relative reflectance spectra.

## Author Contributions

Y.T. and S.Y. wrote the main manuscript text. Y.T., S.Y., M.T., A.T., M.H.R. and T.S. performed the experiments.

## Supplementary Material

Supplementary Information Supplementary Movie 1

Supplementary Information Supplementary Movie 2
